# Light intensity physical activity is beneficially associated with brain volume in older adults with high cardiovascular risk

**DOI:** 10.3389/fcvm.2022.882562

**Published:** 2022-07-13

**Authors:** Keitaro Makino, Sangyoon Lee, Seongryu Bae, Kenji Harada, Ippei Chiba, Osamu Katayama, Kouki Tomida, Masanori Morikawa, Yukari Yamashiro, Motoki Sudo, Naoto Takayanagi, Hiroyuki Shimada

**Affiliations:** ^1^Department of Preventive Gerontology, Center for Gerontology and Social Science, National Center for Geriatrics and Gerontology, Obu, Japan; ^2^Japan Society for the Promotion of Science, Tokyo, Japan; ^3^Tokyo Research Laboratories, Kao Corporation, Tokyo, Japan; ^4^Center for Gerontology and Social Science, National Center for Geriatrics and Gerontology, Obu, Japan

**Keywords:** physical activity, cardiovascular disease, risk score, brain atrophy, community setting

## Abstract

**Background:**

Older people with high cardiovascular risk, including those without cardiovascular diseases, are an at-risk population for dementia. Regular physical activity is generally recommended to maintain brain health; however, the optimal intensity of physical activity for maintaining brain volume in older adults with cardiovascular risk remains unclear. We examined the associations between intensity-specific physical activity and brain volume stratified by absolute cardiovascular risk level in older adults without cardiovascular diseases.

**Methods and results:**

This cross-sectional study involved 725 community-dwelling older Japanese adults without cardiovascular diseases. We estimated absolute cardiovascular risk using the World Health Organization risk estimation charts, which include variables such as age, sex, diabetes mellitus, smoking, systolic blood pressure, and total cholesterol, and stratified cardiovascular risk level into three risk categories: low (≤ 9%), moderate (10–14%), and high (≥15%). We measured daily physical activity using a triaxial accelerometer, and calculated the average time spent in moderate-to-vigorous intensity physical activity (MVPA) and light intensity physical activity (LPA). We performed brain T1-weighted magnetic resonance imaging and calculated the volume of the cortical gray matter, subcortical gray matter, and cerebral white matter, using the FreeSurfer software. In the overall sample, multivariable linear regression analysis showed that greater MVPA was significantly associated with greater volume of the cortical gray matter and cerebral white matter, and greater LPA was significantly associated with greater volume of the cerebral white matter. Additionally, in the analysis of the sample stratified by absolute cardiovascular risk level, cerebral white matter volume was significantly associated with both MVPA and LPA in the high cardiovascular risk group.

**Conclusions:**

The association between physical activity and brain volume differed according to cardiovascular risk level in community-dwelling older adults. In a population at high cardiovascular risk, maintaining or increasing LPA might be a practical and achievable strategy for healthy brain aging.

## Introduction

The prevalence of dementia is rapidly increasing, along with an aging global population. According to predictions, globally, the total number of people with dementia is likely to reach 78 million in 2030, and 139 million in 2050 (1). Thus, numerous studies have been conducted around the world to identify risk factors and effective intervention strategies for maintaining brain health and for preventing dementia.

Cardiovascular risk factors (e.g., diabetes mellitus, smoking, hypertension, and hyperlipidemia) are known to be associated with decreased brain volume in gray matter (2) and white matter (3). Recently, absolute cardiovascular risk level estimated by multivariable risk assessments was advocated to guide treatment of patients with cardiovascular risk factors (4). Previous large-scale cohort studies have developed various cardiovascular risk estimation tools (5–7), and have demonstrated that the estimated cardiovascular risk level is a useful predictor of cognitive decline (8), brain atrophy (9), and white matter hyperintensity (10), as well as cardiovascular disease events. Therefore, older people with high cardiovascular risk, including those without cardiovascular diseases, are considered to be the population more likely to suffer from brain health problems.

Regular physical activity is widely recommended for maintaining brain structure in older age. Tan et al., using the Framingham study cohort data (*n* = 1,987), showed that the amount of physical activity was linearly associated with total cerebral brain and hippocampal volumes (11). Raji et al. (12) examined the effect of change in physical activity on brain structure using the Cardiovascular Health Study data (*n* = 876), and reported that increased physical activity over time was associated with larger gray matter volume. A recent systematic review that included 32 studies also reported that greater physical activity was associated with larger brain volumes in non-demented older adults (13). Based on this evidence, maintenance of daily physical activity has been considered a key factor for brain health in older people.

For healthy older people, the World Health Organization (WHO) recommends moderate-to-vigorous intensity physical activity [MVPA, defined as ≥3.0 metabolic equivalents, such as walking briskly, playing double tennis, or raking the yard (14)] as favorable intensity of physical activity for general health benefits (15). Additionally, a recent systematic review concluded that regular MVPA is beneficial for healthy cognitive aging (16). However, a high-cardiovascular-risk population is considered to have relatively lower physical fitness; therefore, it is necessary to develop an achievable strategy using feasible levels of physical activity, such as light intensity physical activity [LPA, defined as ≥1.6 to <3 metabolic equivalents, such as walking at slow pace, cooking activities, or light household chores (14)].

We identified several previous studies that examined LPA and brain volume in population-based cohorts (17–19). Regarding self-reported LPA, Gu et al. (17) documented that LPA levels assessed by questionnaire were associated with total brain volume, total gray matter, total white matter, and the hippocampus, in 1,443 community-dwelling older adults. Regarding objectively measured LPA using an accelerometer, Spartano et al. (18) reported that LPA was associated with greater brain volume in 2,354 middle and older adults, but this study defined activity intensity by the step counts per unit time, which differs from the typical LPA definition. Machida et al. (19) reported that objectively measured LPA was not associated with brain volume in 485 older adults, but they focused only on the hippocampus volume and its relationship to other brain regions is unknown. Because of these differences in the methods used to measure physical activity and brain volume, the results are not consistent, even among the general population. Therefore, additional examination on the relationship between LPA and brain structure should be conducted using objective, large-scale data.

Moreover, a previous study suggests that LPA as well as MVPA were favorably associated with both cardiovascular risk factors and absolute cardiovascular risk level in older females without cardiovascular diseases (20), and these cardiovascular risk factors are known to be associated with brain health (2, 3). Additionally, Felisatti et al. (21) performed mediation analysis and showed that the relationship between physical activity and brain volume may be mediated by cardiovascular risk factors in cognitively unimpaired older adults. Therefore, we hypothesized that LPA, as well as MVPA, would be beneficially associated with brain volume *via* better control of cardiovascular risk, even in populations at high cardiovascular risk level. We also hypothesized that in those populations LPA may be a practical and achievable strategy for supporting healthy brain aging.

Therefore, we examined the associations between objectively measured intensity-specific physical activity and brain volume of multiple brain regions, in older adults without cardiovascular diseases stratified by absolute cardiovascular risk level.

## Methods

### Study settings and participants

This cross-sectional study involved community-dwelling older Japanese adults, who were enrolled from a sub-cohort of the National Center for Geriatrics and Gerontology-Study of Geriatric Syndromes (NCGG-SGS). The NCGG-SGS is a Japanese national cohort study, the primary aim of which is to establish a screening system for geriatric syndromes and validate evidence-based interventions to prevent them. Overall, 1,220 individuals aged ≥60 years with residence in Takahama City, Aichi prefecture, Japan, completed our health check-up survey and brain magnetic resonance imaging (MRI) assessment. The health check-up included assessments of medical history and sociodemographic information, blood sampling, and physical activity measurement; it was performed by well-trained nurses and study assistants in community centers, and the MRI assessment was undertaken by a radiologist. All staff received training from the authors, in terms of the protocols for administering the assessments prior to study commencement. We applied the following exclusion criteria: (1) history of neuropsychiatric diseases, including dementia and Parkinson's disease (*n* = 6); (2) history of cardiovascular diseases, including stroke and heart diseases (i.e., angina, myocardial infraction, and aortic aneurysm) (*n* = 204); (3) suspected dementia, based on a Mini-Mental State Examination score <21 (22) (*n* = 9); (4) functional disability, based on the requirement of long-term care covered by the insurance system (*n* = 3); (5) refusal to wear an accelerometer or insufficient physical activity data for analysis (*n* = 262); (6) missing data concerning the assessment of absolute cardiovascular risk (*n* = 8); and (7) missing data in any of the above criteria (*n* = 3). After exclusions, the data of 725 participants were available for analysis.

### Ethical approval

The study protocol was developed in accordance with the Helsinki Declaration and was approved by the Ethics Committee of the National Center for Geriatrics and Gerontology (Approval Number: 1440-3). We disclosed information about the study using the opt-out approach, and the data of study subjects who declined study participation directly or *via* proxy were excluded.

### Estimation of absolute cardiovascular risk

We estimated 10-year cardiovascular disease risk using the revised WHO risk estimation charts (2019) (23). These estimation charts indicate the absolute risk of a cardiovascular disease event according to an individual's risk status, with a higher risk score indicating a greater risk-factor burden. The development group of the estimation charts calibrated prediction models for 21 global regions, and region-specific prediction charts were presented. The estimation charts provide two types of estimation models: a laboratory-based model including medical history and blood data, and a non-laboratory-based model that consists of convenient variables for resource-limited settings. In this study, we used the laboratory-based risk estimation model that includes age, sex, diabetes mellitus, smoking status, systolic blood pressure, and total cholesterol, for the high-income Asia-Pacific region, including Japan (23).

Regarding each component of the WHO risk estimation model, we assessed diabetes mellitus, smoking status, systolic blood pressure, and total cholesterol levels, along with age and sex. Current status of diabetes mellitus was assessed through face-to-face interviews by nurses. Nurses measured systolic blood pressure using an automated sphygmomanometer, with participants in a seated position. Total serum cholesterol levels (in mmol/L) were measured by enzyme-based methods at a laboratory (Good Life Design Corporation, Japan). Smoking status was assessed as the presence or absence of regular smoking (current vs. former/never) by the study assistants. Finally, we calculated absolute cardiovascular risk (%), based on the above risk status, using the revised WHO risk estimation charts, and stratified cardiovascular risk into three levels, namely, low (≤ 9%), moderate (10–14%), and high (≥15%).

### Measurement of physical activity

Daily physical activity was measured using a triaxial accelerometer (WH-100; Kao Corporation, Japan) with a 4-s epoch length. The intensity level of an activity was calculated according to the algorithms of the Kenz Lifecorder (Suzuken Corporation, Japan) (24), on a scale of 0.5 (minimal intensity of movement) to 9 (maximal intensity of movement). Participants were asked to wear the waist accelerometer all day, apart from when they were bathing or sleeping. We considered accelerometer data valid when participants wore the device for ≥10 h (25) a day, and for at least 7 days of the initial 14 days (26). We calculated the average time spent in MVPA and LPA (min/day) for each participant. In this study, MVPA and LPA were defined as intensity levels 4–9 (≥3.0 metabolic equivalents) and intensity levels 1–3 (≥1.6 to <3 metabolic equivalents), respectively, according to the previous validation study (24) and the current guidelines of physical activity (14).

### MRI acquisition and image processing

Structural brain MRI was performed using a Siemens MAGNETOM Trio 3T scanner (Siemens Medical Solutions, Germany), using a 12-channel head coil. A three-dimensional T1-weighted magnetization allowed the rapid acquisition of weighted images in a gradient echo sequence using the following parameters: repetition time = 1,800 ms; echo time = 1.99 ms; flip angle = 9°; matrix = 256 × 256; field of view = 250 mm; voxel size = 1 × 1 × 1.1 mm; slice thickness = 1.1 mm; slices = 160 sagittal slices; and acquisition time = 4:06 min (27).

We used FreeSurfer version 7 (http://surfer.nmr.mgh.harvard.edu) running on a Linux server (Ubuntu version 20.04) for image processing (28). The automated processing stream mainly included the removal of non-brain tissue (29), Talairach transformation, segmentation of gray/white matter tissue (30), intensity normalization, topological correction of the cortical surface (31), and surface deformation for optimal placement of the tissue borders (32). We calculated brain volumetric measurements (mm^3^) including those of cortical gray matter (CGM), subcortical gray matter (SGM), and cerebral white matter (CWM).

### Potential confounding factors

As covariates, information on age, sex, education level, and medical history of depression, cancer, and pulmonary disease (i.e., pneumonia, tuberculosis, and chronic obstructive pulmonary disease), was obtained. Education level and medical history were assessed through face-to-face interviews. Moreover, we included slow gait speed and depressive symptoms as covariates. Gait speed was measured over a 2.4-m distance, and the mean gait speed of five trials under 1.0 m/s was defined as a slow gait speed (33). Depressive symptoms were assessed using the 15-item Geriatric Depression Scale, and participants who scored 6 or higher were considered to have depressive symptoms (34).

### Statistical analysis

Participants' characteristics were compared according to cardiovascular risk levels (low, moderate, and high) using one-way analysis of variance for continuous variables and χ^2^ test for categorical variables. Further, we compared brain volume (CGM, SGM, and CWM) according to cardiovascular risk levels using analysis of covariance adjusted for total cranial volume with Bonferroni corrected *post-hoc* comparison.

We conducted multivariable linear regression analysis to examine the association between physical activity and brain volume. We applied this model to the overall sample and to the cardiovascular risk-stratified sample (low, moderate, and high-risk group). In these models, physical activity as an independent variable was categorized into quartiles (Q1, low; Q2, mid-low; Q3, mid-high; Q4, high), and β with 95% confidence interval (CI) in each physical activity category was calculated in the crude and adjusted models. Regarding covariates, estimated total cranial volume and other characteristics that had significant association with brain volume (either of CGM, SGM, or CWM) were controlled in the adjusted models. Correction for multiple testing was performed with the Benjamini-Hochberg procedure, with a false discovery rate set to 0.05 (35).

All analyses were performed using IBM SPSS Statistics 25 (IBM Japan, Tokyo, Japan) software. The level of statistical significance was set to *P* < 0.05.

## Results

### Participants' characteristics

Of 725 participants without cardiovascular diseases, group classification according to estimated cardiovascular risk level was as follows: low risk group, *n* = 222 (30.6%); moderate risk group, *n* = 269 (37.1%); high risk group, *n* = 234 (32.3%). Differences in participant characteristics between the three cardiovascular risk groups are shown in Table 1. Except for total cholesterol (*P* = 0.809), there were significant differences in all components of the WHO risk estimation model: age (*P* < 0.001), sex (*P* < 0.001), prevalence of diabetes mellitus (*P* < 0.001), proportion of current smokers (*P* < 0.001), and systolic blood pressure (*P* < 0.001). In addition, there were significant differences in education level (*P* = 0.002) and in the prevalence of cancer (*P* = 0.030) between participants with different cardiovascular risk levels.

**Table 1:** Participant characteristics according to estimated cardiovascular risk levels.

			**Cardiovascular risk levels**			
		**Overall *n* = 725**	**Low-risk group (≤ 9%) *n* = 222**	**Moderate-risk group (10–14%) *n* = 269**	**High-risk group (≥15%) *n* = 234**	***P***
**Components of WHO risk estimation model**						
Age	(years)	69.6 ± 6.0	65.4 ± 4.1	70.3 ± 5.8	72.9 ± 5.5	<0.001
Female	(*n*, %)	379 (52.3)	186 (83.8)	148 (55.0)	45 (19.2)	<0.001
Diabetes mellitus	(*n*, %)	68 (9.4)	3 (1.4)	13 (4.8)	52 (22.2)	<0.001
Smoking status	(*n*, %)	74 (10.2)	5 (2.3)	22 (8.2)	47 (20.1)	<0.001
Systolic blood pressure	(mmHg)	140.0 ± 19.7	128.5 ± 16.4	138.9 ± 16.9	152.3 ± 18.7	<0.001
Total cholesterol	(mmol/L)	5.6 ± 0.9	5.6 ± 0.9	5.6 ± 0.9	5.6 ± 0.8	0.809
**Other characteristics**						
Education level	(years)	11.8 ± 2.4	12.3 ± 2.2	11.6 ± 2.3	11.7 ± 2.6	0.002
Depression	(*n*, %)	18 (2.5)	6 (2.7)	10 (3.7)	2 (0.9)	0.116
Cancer	(*n*, %)	74 (10.2)	18 (8.1)	22 (8.2)	34 (14.5)	0.030
Pulmonary disease	(*n*, %)	60 (8.3)	24 (10.8)	20 (7.4)	16 (6.8)	0.251
Slow gait speed	(*n*, %)	76 (10.5)	17 (7.7)	27 (10.2)	32 (13.7)	0.104
Depressive symptoms	(*n*, %)	71 (9.8)	21 (9.5)	27 (10.1)	23 (9.8)	0.974

*WHO, World Health Organization.*

*Data are expressed as mean ± standard deviation or numbers (%).*

*P-values are based on one-way analysis of variance for continuous variables and χ^2^ tests for categorical variables*.

**Figure 1:**
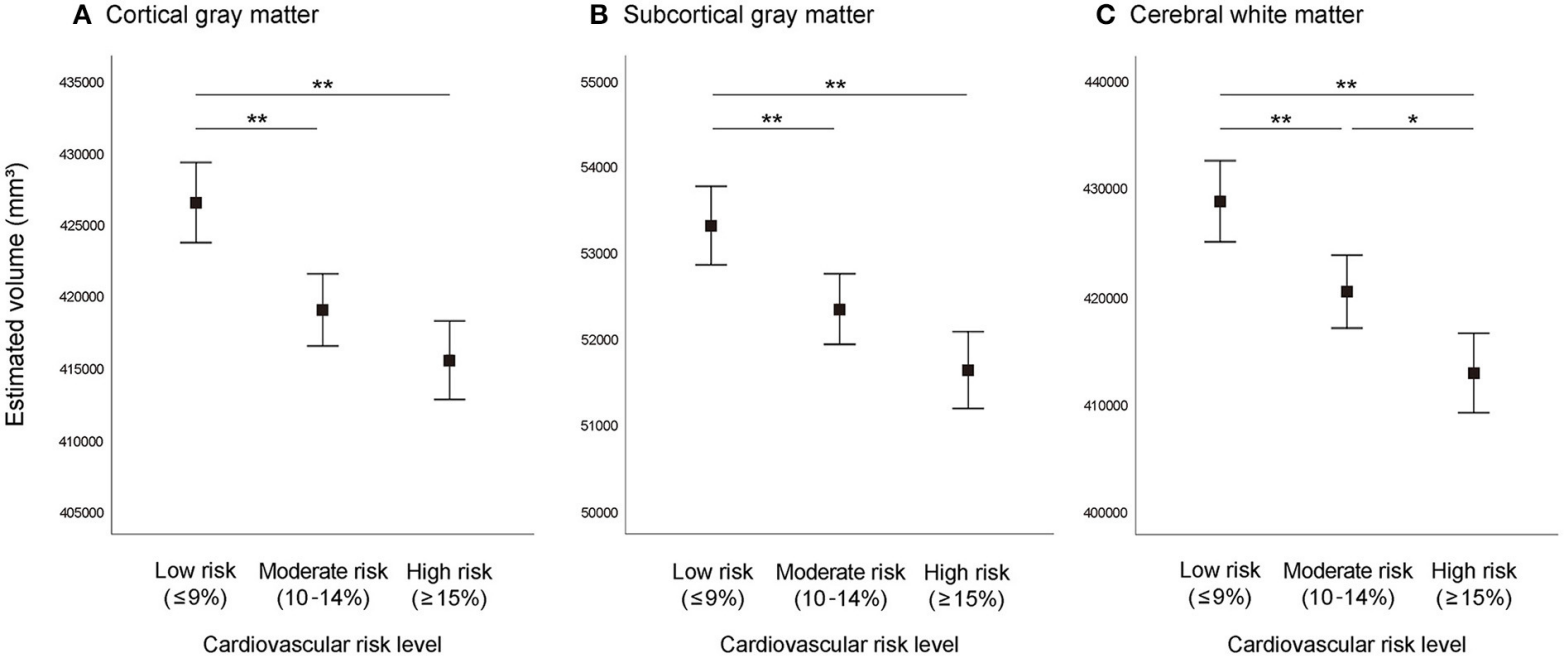
Brain volume according to absolute cardiovascular risk level. **(A)** Cortical gray matter, **(B)** subcortical gray matter, **(C)** cerebral white matter. Graphs represent estimated mean values and 95% confidence intervals adjusted for total cranial volume. ^**^P < 0.01, ^*^P < 0.05.

Differences in brain volume between the three cardiovascular risk groups adjusted for total cranial volume are shown in Figure 1. Analysis of covariance showed significant differences (*P* < 0.001) of CGM, SGM, and CWM between the three cardiovascular risk groups. In multiple comparisons, compared with the moderate and high cardiovascular risk groups, the low cardiovascular risk group showed significantly greater (*P* < 0.01) brain volume of CGM, SGM, and CWM. Similarly, the moderate cardiovascular risk group showed significantly greater volume of CWM compared to the high cardiovascular risk group (*P* < 0.05).

### Associations between physical activity and brain volume in the overall sample

**Figure 2:**
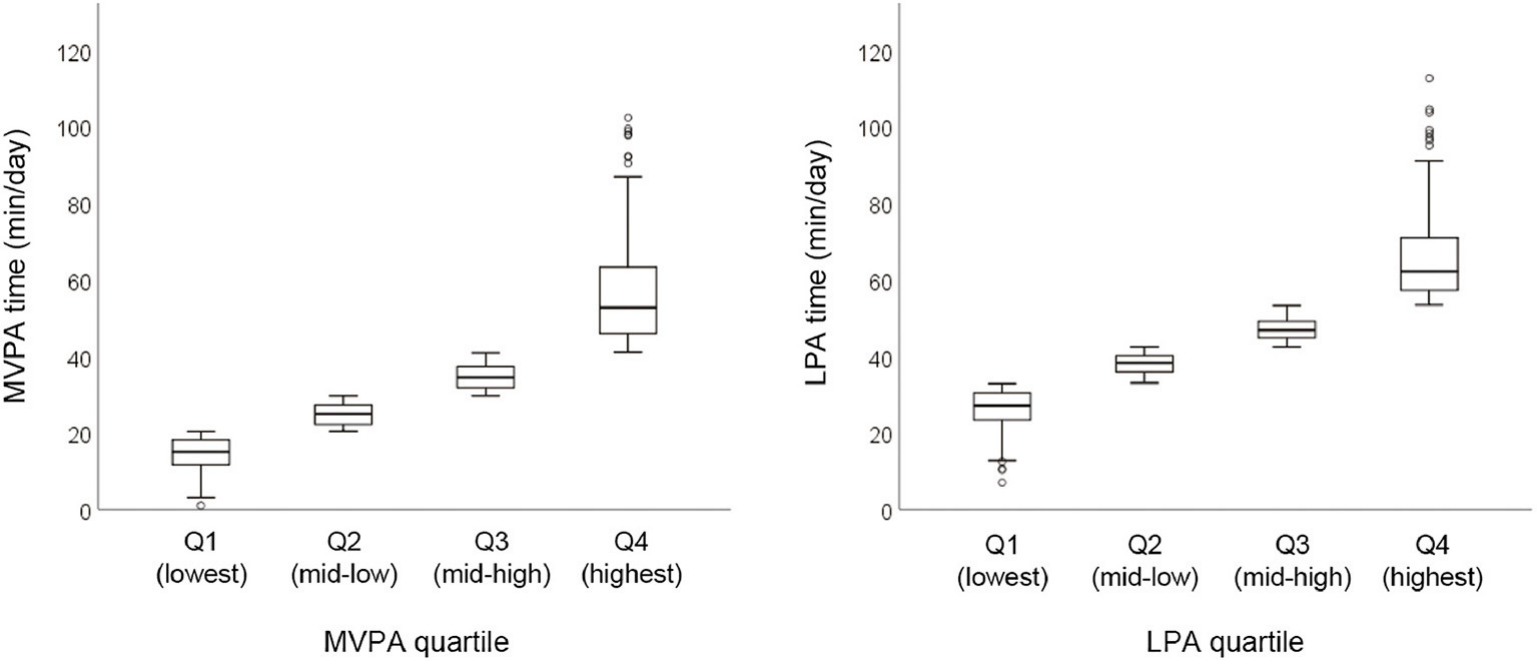
Physical activity time spent in MVPA and LPA in the overall sample. Graphs represent median and range per quartile. MVPA, moderate- to vigorous-intensity physical activity; LPA, low-intensity physical activity; Q, quartile of physical activity.

The median and range of time (min/day) spent in MVPA and LPA per quartile were as follows: Q1 = 15.1 (1.0–20.3), Q2 = 24.9 (20.4–29.7), Q3 = 34.5 (29.8–40.9), and Q4 = 52.7 (41.0–102.3) in MVPA, and Q1 =27.1 (7.0–32.9), Q2 = 38.3 (33.0–42.4), Q3 =46.8 (42.5–53.3), and Q4 =62.2 (53.4–112.6) in LPA (Figure 2).

The results of the linear regression analysis in the overall sample (*n* = 725) are shown in Table 2. In the adjusted models, greater MVPA was significantly associated with greater brain volume in CGM (compared to the lowest MVPA quartile, β of the highest MVPA quartile = 0.06, 95% CI = 0.01–0.10, and P for trend = 0.041) and CWM (compared to the lowest MVPA quartile, β of the highest MVPA quartile = 0.06, 95% CI = 0.01–0.11, and P for trend = 0.021), however, this trend was not significant for SGM. Furthermore, greater LPA was significantly associated with greater brain volume in CWM (compared to the lowest LPA quartile, β of the highest LPA quartile = 0.09, 95% CI = 0.04–0.14, and P for trend = 0.009), however, this trend was not significant for CGM and SGM.

**Table 2:** Physical activity and brain volume in the overall sample.

			Brain volumes*	**Crude model**		**Adjusted model**	
**Brain measures**	**Physical activity**		**mm** ^3^	**β**	**95% CI**	β^*^	**95% CI**
CGM volume	MVPA quartile	Q1 (lowest)	417,131.8	Ref		Ref	
		Q2 (mid-low)	420,426.4	0.08	−0.01 to 0.17	0.04	−0.01 to 0.09
		Q3 (mid-high)	421,042.0	0.09	0.01 to 0.18	0.05	0.00 to 0.09
		Q4 (highest)	421,894.3	0.20	0.11 to 0.29	0.06	0.01 to 0.10
	*P* for trend			<0.001		0.041	
	LPA quartile	Q1 (lowest)	417,731.5	Ref		Ref	
		Q2 (mid-low)	419,776.2	0.05	−0.04 to 0.14	0.02	−0.02 to 0.07
		Q3 (mid-high)	421,104.1	−0.01	−0.10 to 0.08	0.04	−0.01 to 0.09
		Q4 (highest)	421,879.4	0.07	−0.02 to 0.16	0.05	0.00 to 0.10
	*P* for trend			0.263		0.065	
SGM volume	MVPA quartile	Q1 (lowest)	51,921.3	Ref		Ref	
		Q2 (mid-low)	52,433.7	0.10	0.01 to 0.19	0.05	−0.01 to 0.10
		Q3 (mid-high)	52,952.3	0.15	0.06 to 0.24	0.09	0.03 to 0.15
		Q4 (highest)	52,300.1	0.17	0.09 to 0.26	0.03	−0.03 to 0.09
	*P* for trend			<0.001		0.121	
	LPA quartile	Q1 (lowest)	52,053.7	Ref		Ref	
		Q2 (mid-low)	52,497.8	0.08	−0.01 to 0.17	0.04	−0.02 to 0.10
		Q3 (mid-high)	52,425.1	0.01	−0.08 to 0.10	0.03	−0.03 to 0.09
		Q4 (highest)	52,629.9	0.10	0.01 to 0.19	0.05	−0.01 to 0.11
	*P* for trend			0.159		0.133	
CWM volume	MVPA quartile	Q1 (lowest)	416,636.7	Ref		Ref	
		Q2 (mid-low)	419,238.9	0.07	−0.02 to 0.16	0.02	−0.03 to 0.07
		Q3 (mid-high)	422,547.9	0.10	0.02 to 0.19	0.05	0.00 to 0.10
		Q4 (highest)	423,525.9	0.21	0.12 to 0.29	0.06	0.01 to 0.11
	*P* for trend			<0.001		0.021	
	LPA quartile	Q1 (lowest)	415,578.0	Ref		Ref	
		Q2 (mid-low)	421,938.3	0.09	0.00 to 0.18	0.06	0.01 to 0.10
		Q3 (mid-high)	418,903.8	−0.01	−0.10 to 0.08	0.03	−0.02 to 0.08
		Q4 (highest)	425,535.2	0.12	0.03 to 0.21	0.09	0.04 to 0.14
	*P* for trend			0.216		0.009	

*CI, confidence interval; CGM, cortical gray matter; SGM, subcortical gray matter; CWM, cerebral white matter; MVPA, moderate- to vigorous-intensity physical activity; LPA, low-intensity physical activity; Q, quartile of physical activity as an independent variable.*

^*^Adjusted for age, sex, education level, and total cranial volume.

*All P values are corrected for multiple testing using the Benjamini-Hochberg with false discovery rate set to 0.05*.

### Associations between physical activity and brain volume stratified by absolute cardiovascular risk levels

**Figure 3:**
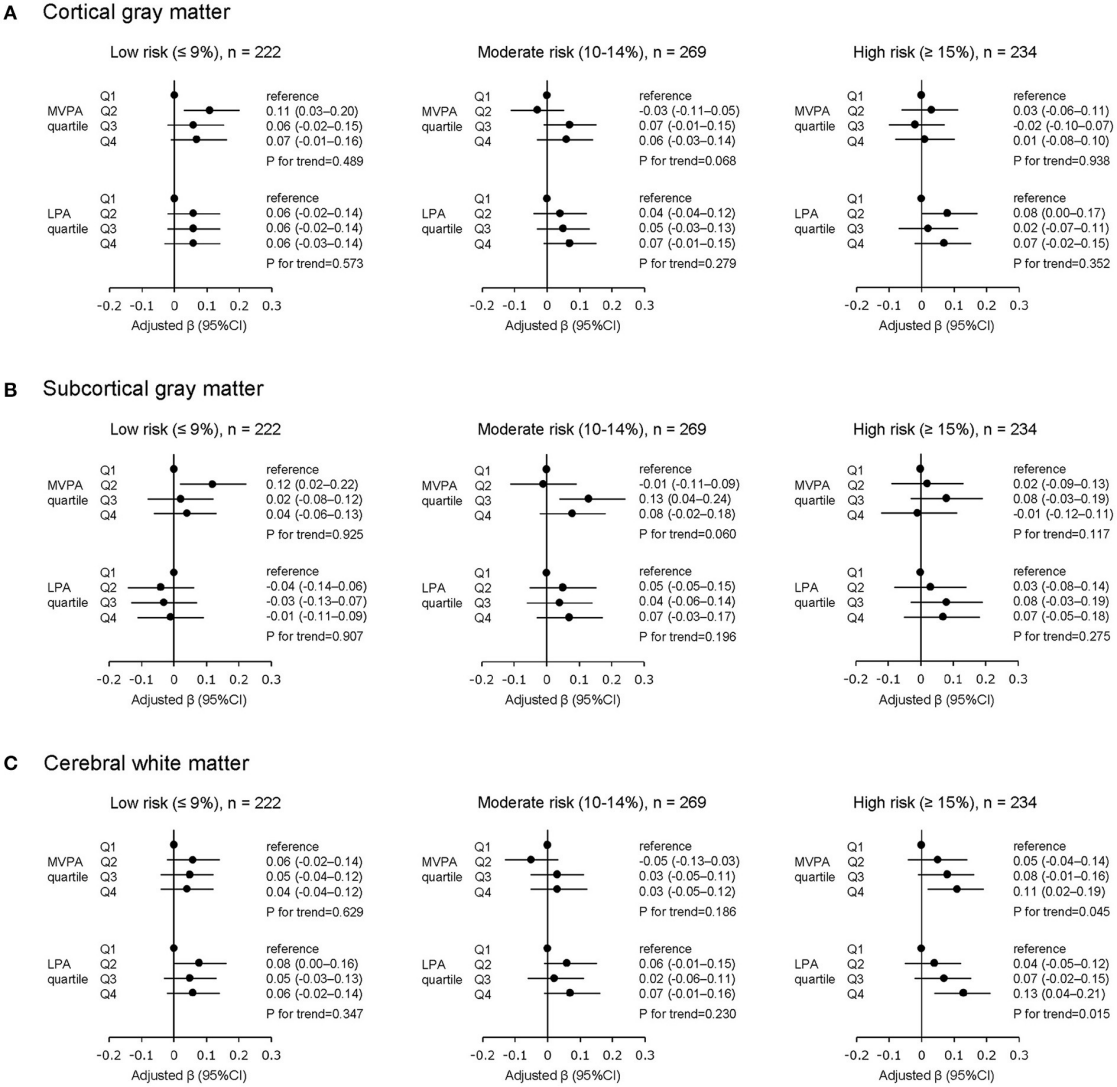
Physical activity and brain volume stratified by absolute cardiovascular risk levels. **(A)** Cortical gray matter, **(B)** subcortical gray matter, **(C)** cerebral white matter. MVPA, moderate- to vigorous-intensity physical activity; LPA, low-intensity physical activity; Q, quartile of physical activity as an independent variable; CI, confidence interval. All β values are adjusted by age, sex, education level, and total cranial volume. All *P* values are corrected for multiple testing using the Benjamini-Hochberg with false discovery rate set to 0.05.

The results of the multivariable linear regression analysis in the cardiovascular risk-stratified sample are shown in Figure 3. Among participants in the low (*n* = 222) and moderate (*n* = 269) cardiovascular risk groups there was no significant trend between physical activity (both MVPA and LPA) and brain volume in all brain measures.

Among participants in the high cardiovascular risk group (*n* = 234), greater MVPA was significantly associated with greater volume in CWM (compared to the lowest MVPA quartile, β of the highest MVPA quartile = 0.11, 95% CI = 0.02–0.19, and P for trend = 0.045), but not with volume in CGM and SGM. Furthermore, similar to the MVPA results, greater LPA was significantly associated with greater volume in CWM (compared to the lowest LPA quartile, β of the highest LPA quartile = 0.13, 95% CI = 0.04–0.21, and P for trend = 0.015), but not with volume in CGM and SGM.

## Discussion

This cross-sectional study indicated that among community-dwelling older adults greater MVPA was significantly associated with greater volume in CGM and CWM, and greater LPA was significantly associated with greater volume in CWM. Additionally, in the stratified analysis using absolute cardiovascular risk levels, the high cardiovascular risk group showed a significant relationship between physical activity and CWM volume, but the low and moderate cardiovascular risk groups did not. These results suggested that the association between physical activity and brain volume differed according to cardiovascular risk level among community-dwelling older adults.

We confirmed the relationship between cardiovascular risk level and brain volume, and demonstrated that the moderate and high cardiovascular risk groups have smaller volume in CGM, SGM, and CWM, compared to the low cardiovascular risk group. Song et al. analyzed data from a large cohort of older adults (*n* = 394) and showed that higher absolute cardiovascular risk was associated with smaller volume in CGM and SGM, but not in CWM (9). Santos et al. (36) conducted voxel-wise morphometric MRI study of older adults (*n* = 156) and reported that, compared to the low and moderate cardiovascular risk groups, the high cardiovascular risk group showed smaller white matter volumes in the prefrontal-parietal region. Our results are consistent with these findings. Moreover, our findings, which are based on the analysis of a larger sample, support the existing evidence on the relationship between cardiovascular risk level and brain volume.

In the overall sample, we found a statistically significant trend for increasing brain volume in CGM and CWM with higher MVPA, and we also found a statistically significant trend for increasing CWM volume with higher LPA. Although the benefits of MVPA for cognitive health are widely known (16), the impact of LPA has been unclear. The results of previous studies which examined LPA and brain volume in population-based cohorts are inconsistent (18, 19), which may be due to differences in the definition or methods used to measure LPA and brain volume. Machida et al. calculated time of MVPA and LPA using the same definition as ours and reported that MVPA was associated with hippocampal volume but LPA was not (19); however, they did not examine the association between LPA and other brain regions beyond the hippocampus. We conducted a large scale cohort study with objectively measured physical activity and multiple brain volume indices, and our study's finding that LPA as well as MVPA is associated with brain volume seems valuable. Although the underlying mechanisms remain to be elucidated, physical activity is regarded to have a protective effect on the brain through several pathways (37). One of the major mechanisms is the increase of brain-derived neurotrophic factor (BDNF) in the blood. BDNF is considered to support brain preservation by mediating neuronal survival and neurogenesis, and it may also preserve white matter structure (38). However, although there have been numerous studies on this topic, it is not fully understood how blood BDNF induced by physical activity actually preserves brain structure (37). Physical activity is also supposed to protect brain volume through anti-neurodegenerative mechanisms. Regular physical activity is considered to regulate the cellular redox state of the brain, and this antioxidative effect may support neuroprotection (39). In addition, physical activity also helps reduce proinflammatory conditions such as obesity and insulin disturbances (21). These anti-inflammatory effects may counteract neurodegeneration.

This study is novel because it included the analysis of subjects stratified by their absolute cardiovascular risk level. In the present study regarding the differences in the association of physical activity and brain structure between patients with different cardiovascular risk levels, the high (≥15%) cardiovascular risk group showed a significant relationship between physical activity and brain volume in CWM after adjusting for covariates, while the low ( ≤ 9%) and moderate (10–14%) cardiovascular risk group showed no significant trend between physical activity and brain volume. This selective association between physical activity and brain volume only in the high cardiovascular risk group may be explained by the different pathways of protective effects physical activity exerts on the brain. As described above, physical activity directly induces the release of neurotrophic factors, and activates neurogenesis and synaptic plasticity that independently protect the brain. On the other hand, physical activity also reduces inflammation induced by cardiovascular risk factors, which in turn indirectly improves growth factor signaling and mitigates their adverse effects on the brain (21). Taking these into account, the high cardiovascular risk group in this study may be in a relatively elevated inflammatory state and may be more likely to benefit from the anti-inflammatory effects of physical activity. Thus, the association between physical activity and brain volume in the high cardiovascular risk group may have been emphasized by effect modification through an indirect pathway. In terms of why physical activity was associated with CWM in the high cardiovascular risk group and not with CGM and SGM, higher cardiovascular risk levels are known to be associated with more advanced white matter lesions (10), and the white matter volume in the high cardiovascular risk groups may also be influenced by white matter hyperintensity. Physical activity has a beneficial effect on both white matter structure and white matter lesions (40), thus the association with physical activity may have been detected only in CWM in our stratified analysis. Moreover, individuals with severe white matter lesions are at more risk of mobility (41) or gait dysfunction (42). This negative effect on physical function could lead to their lower engagement in physical activity, and may be another explanation for the association highlighted only in CWM in the high cardiovascular risk group.

Our most clinically meaningful finding is that brain volume was significantly associated with not only MVPA but also with LPA, after adjusting for covariates in the high cardiovascular risk group. To the best of our knowledge, this result provides the first objective evidence of the beneficial association of LPA with brain volume in a population with a relatively high absolute cardiovascular risk. Although our cross-sectional analysis cannot demonstrate the causal relationship between LPA and brain volume, maintaining or increasing LPA might be a practical and achievable strategy for healthy brain aging in older people at high cardiovascular risk. Additionally, LaMonte et al. (20) reported that greater LPA as well as MVPA were associated with lower cardiovascular risk. Therefore, our results may be helpful for halting the accelerated increase in dementia risk due to cardiovascular dysfunction. Further studies are required to establish effective intervention strategies based on daily physical activity including LPA, for older adults at high cardiovascular risk.

### Strengths and limitations

The major strength of this study lies in it examining the associations between intensity-specific physical activity and brain volume in a population that was stratified according to absolute cardiovascular risk level. Additionally, we analyzed data from a large and well-characterized cohort, including data on structural brain MRI and objectively measured physical activity. Measuring physical activity using an accelerometer, rather than a self-reported questionnaire, is known to reduce the chance of recall or social desirability bias (43). Moreover, since we analyzed population-based data of older adults without cardiovascular diseases at baseline, our findings can be generalized to community-dwelling people in primary care settings.

However, our study had some limitations. First, this study included a cross-sectional design; thus, it cannot show causal relationships between physical activity, brain structure, and cardiovascular fitness. Second, we did not measure biomarkers related to the prognosis of Alzheimer's pathology, such as apolipoprotein E genotype or amyloid status; therefore, we cannot confirm the pathological mechanisms between physical activity and brain health. Additional longitudinal studies including time series data of daily physical activity and biomarkers that reflect brain pathology are required to confirm the causal relationship between physical activity and brain health in older adults with cardiovascular risk. Third, although we did not address subregions of the brain in this study, a previous study has shown that different brain subregions have different sensitivity to the effects of age-related changes (44). Therefore, in the future, it is necessary to verify our findings in each subregion of the brain.

## Conclusion

Our large-scale cohort study demonstrated that greater physical activity was associated with greater brain volume, regardless of physical activity intensity, among community-dwelling older adults. Moreover, we found that LPA was beneficially associated with CWM volume in older adults with high cardiovascular risk. Maintaining or increasing LPA might be a practical and achievable strategy for ensuring healthy brain aging in a high-cardiovascular-risk population.

## Data availability statement

The datasets presented in this article are not readily available because participants of this study did not agree for their data to be shared publicly. Requests to access the datasets should be directed to KM, kmakino@ncgg.go.jp.

## Ethics statement

The studies involving human participants were reviewed and approved by Ethics Committee of the National Center for Geriatrics and Gerontology. Written informed consent for participation was not required for this study in accordance with the national legislation and the institutional requirements.

## Author contributions

KM designed the study, analyzed and interpreted the data, and wrote and edited the manuscript. HS administered the project, acquired funding for the study, and reviewed and edited the manuscript. SL, SB, and KH contributed to the acquisition, analysis, and interpretation of data and reviewed and edited the manuscript. YY, MS, and NT contributed to the acquisition of data and reviewed and edited the manuscript. IC, OK, KT, and MM contributed to the formulation of the discussion and reviewed and edited the manuscript. All authors contributed to the article and approved the submitted version.

## Funding

This work received financial support *via* JSPS KAKENHI grant-in-aid for Scientific Research A (26242059), for JSPS Research Fellow (20J01647), and for Early-Career Scientists (20K19442), Research Funding for Longevity Sciences from the National Center for Geriatrics and Gerontology (27-22), and funds from Takahama City Local Government. Additionally, this work was financially supported as a joint research with Kao Corporation.

## Conflict of interest

Authors YY, MS, and NT were employed by the Kao Corporation. HS received grant funding from the Kao Corporation. The funder had the following involvement: data collection and preparation of the manuscript. The remaining authors declare that the research was conducted in the absence of any commercial or financial relationships that could be construed as a potential conflict of interest.

## Publisher's note

All claims expressed in this article are solely those of the authors and do not necessarily represent those of their affiliated organizations, or those of the publisher, the editors and the reviewers. Any product that may be evaluated in this article, or claim that may be made by its manufacturer, is not guaranteed or endorsed by the publisher.
